# Linkage analysis of systolic blood pressure: a score statistic and computer implementation

**DOI:** 10.1186/1471-2156-4-S1-S77

**Published:** 2003-12-31

**Authors:** Kai Wang, Yingwei Peng

**Affiliations:** 1 Department of Biostatistics, Division of Statistical Genetics, The University of Iowa, Iowa City, Iowa, USA; 2 Department of Mathematics and Statistics, Memorial University of Newfoundland, St. John's, Newfoundland A1C 5S7, Canada

## Abstract

A genome-wide linkage analysis was conducted on systolic blood pressure using a score statistic. The randomly selected Replicate 34 of the simulated data was used. The score statistic was applied to the sibships derived from the general pedigrees. An add-on R program to GENEHUNTER was developed for this analysis and is freely available.

## Background

Recently, score statistics have been proposed for mapping quantitative trait loci (QTLs) using sibship data or general pedigree data [1-4]. These score statistics are straightforward to compute. These works focus mainly on the development of the methodology. In this report, we apply a score statistic to sibship data derived from the randomly selected Replicate 34 of the simulated data provided by the Genetic Analysis Workshop 13 (GAW13).

The statistic to be used is the one in Wang and Huang [2] that assumes no locus-specific dominance effect. Using a parameterization first introduced by Tang and Siegmund [1], Wang [4] introduced two score statistics to detect QTLs with general pedigrees. One assumes no locus-specific dominance effect and the other one does not. Since the approach in Wang [4] is more general, and since, for sibship data, the two statistics in Wang and Huang [2] and Wang [4] that assume no locus-specific dominance effect are equivalent, we first briefly describe the statistic in Wang [4]. A genome scan was conducted using this statistic. For this purpose, an add-on R program to GENEHUNTER was developed. This R program is freely available.

## Methods

### The score statistic

Let *y*_*k *_and *y*_*l *_be the phenotypic values of individuals *k *and *l*, respectively, in a pedigree. It is assumed that there is no locus-specific dominance effect. Then conditional on the number of alleles shared identical by descent (IBD) by *k *and *l *at a putative locus *t*, we have [4]



where Φ_*kl *_is the kinship coefficient between individual *k *and individual *l*,  is the additive variance due to locus *t*, and 1_{τ=*k*} _is the indicator function which equals 1 when τ = *k *and equals 0 otherwise. The first term, *Cov*(*y*_*k*_,*y*_*l*_), is completely determined by the segregation parameters, such as means, the total additive variance, the total dominance variance, the variance of environmental effect, etc. These parameters can be estimated beforehand using phenotypic data only. The remaining term depends only on the pedigree structure through Φ_*kl *_and the linkage parameter (i.e., ). The hypotheses of interest are



Under the null hypothesis, the segregation parameters are independent of the linkage parameter [1], a property useful for deriving the score statistic. Let *n*_*i *_be the number of individuals in pedigree *i *and  be a vector of phenotypic values in pedigree *i *whose mean is denoted by **μ**_*i*_. The mean **μ**_*i *_may depend on other covariates in a linear or nonlinear fashion [5]. Define **Σ**_*i*0 _= (*Cov*(*y*_*ik*_,*y*_*il*_)), , and



where  and  are the vectors of the probabilities that IBD = 1 and 2, respectively, for all the relative pairs in pedigree *i*. The score statistic, denoted by *S*, is defined as *S *= *b*^2^/*Var*(*b*) if *b *> 0; *S *= 0 otherwise, where *b *= Σ_*i *_(**π**_*i *_- E(**π**_*i*_))^*t*^(**v**_*i *_- E(**v**_*i*_)) and, for sibship data, *Var*(*b*) = *Var*(π)Σ_*i*_Σ_*k*<*l*_*Var*(*w*_*ik*_*w*_*il*_). See Wang and Huang [2] and Wang [4] for more discussion on the calculation of *b *and *Var*(*b*). The score statistic *S *is asymptotically distributed as 0.5 χ_0_^2 ^+ 0.5 χ_1_^2 ^[1,4]. For sibship data, *S *is equivalent to the statistic in [2] that assumes no dominance effect.

### Data analysis

Replicate 34 of the simulated data was randomly chosen and a genome scan was done using the statistic *S *defined above. Systolic blood pressure (SBP) was used as the phenotype. To generate a single value of SBP from its repeated measurements for an individual, SBP was regressed over body mass index (BMI) and hypertensive treatment for each individual using the repeated measurements. The intercept of this individual-wise regression was used as the phenotype of the corresponding individual. We did not adjust the SBP further across different individuals using other covariates such as age, gender, or antihypertensive treatment that are believed to influence SBP, as pointed out by two anonymous reviewers. The power of our analysis may thus be hampered. Both Cohort 1 and Cohort 2 data were used.

The genome scan was facilitated by an R [6] program. This R program requires two input files, one is a phenotype file that contains the pedigree structure and the phenotypic data for each individual, and the other one is a file containing the IBD sharing probabilities exported from GENEHUNTER (Version 2.1 r3) [7,8] by using command dump ibd. At each scanned location, this R program generates the value of the score statistic *S *described in this report and the associated *p*-value computed based on the asymptotic distribution 0.5 χ_0_^2 ^+ 0.5 χ_1_^2^, which is *p*-value = 0.5*Pr*(χ_1_^2 ^≥ *c*), where *c *is the value of the score statistic *S*. This R program dissects a pedigree into nuclear families that are treated as if they were biologically unrelated. The sibship data from all nuclear families were used for the analysis.

One reason for us to consider sibships instead of general pedigrees is that GENEHUNTER outputs IBD sharing probabilities for common relative pairs (including sib-pairs) only. For some pedigrees that are beyond GENEHUNTER's capacity, there is no output on IBD sharing probabilities for any relative pairs in such pedigrees. Such pedigrees are therefore not included in our analysis. In the R program, the mean of the phenotype is empirically estimated by its sample mean for all sibs in all sibships (so it is the same for each sib). The mean and variance of *w*_*ik*_*w*_*il *_are also empirically estimated. See Wang [2] for details.

This R program used for the analysis can be freely downloaded from either  or . The source code is commented. It is expected that its usage be clear from the comments and this report. No separate documentation is provided.

## Results

### Genome scan results

**Figure 1:**
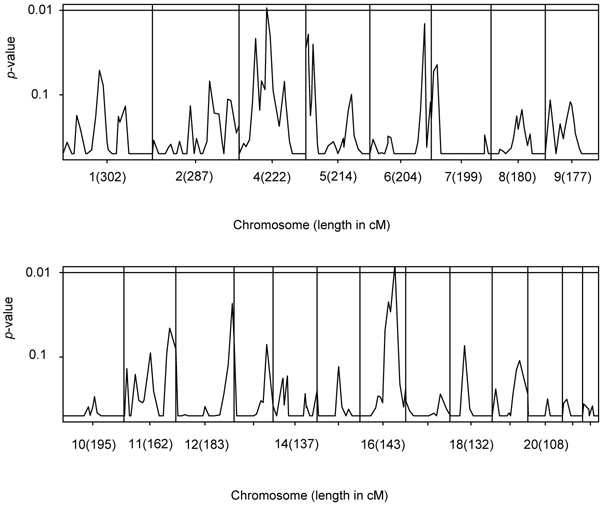
*p*-Values for the score statistic throughout the genome

The score statistic is calculated throughout the genome for the adjusted SBP. The *p*-value at each marker location is graphed in Figure 1. Chromosome 3 is excluded from the analysis because, for this chromosome, the number of markers on the marker map provided by GAW13 is not consistent with that in the corresponding pedigree file.

There is no strong linkage signal from our analysis. There are two markers whose *p*-value is smaller than, but very close to 0.01: one is marker c4g10 (106.13 cM) on chromosome 4 and the other one is marker c16g11 (109.60 cM) on chromosome 16.

## Conclusions

We applied a score statistic to map QTLs that influence SBP using a randomly selected replicate. This score statistic is asymptotically equivalent to the likelihood ratio statistic and is straightforward to calculate. An R program was developed to facilitate the calculation of the score statistic using output on IBD sharing probabilities from GENEHUNTER. Although the score statistic is applicable to general pedigrees, we considered only sibships partly due to the fact that the IBD sharing probabilities from GENEHUNTER are available for common relative pairs (including sib pairs) only.

No significant linkage signal was found. There were two markers whose *p*-values were less than 0.01 (Figure 1). There are several possible causes to our failure to find any significant linkage signal. The replicates chosen may not contain any strong linkage signal. Or the way the phenotype data were preprocessed was not appropriate. For example, we did not consider the effect of age and gender. As far as we know, how to incorporate covariates into the linkage analysis of hypotension is an active field of research [9-11]. We also suspect the use of sibship data, instead of the pedigree data, reduces the power to detect linkage.

As mentioned earlier, the R program developed for this analysis handles sibships only. General pedigrees are dissected into sibships first. We are considering expanding its capacity to be able to handle nuclear families so the phenotypic information on the parents can be incorporated into the analysis. Because the calculation of IBD sharing probabilities for an arbitrary relative pair remains difficult, even using the MCMC technique, implementing the score statistic for large pedigrees does not seem to be simple at this moment.

The size of the output file on IBD sharing probabilities from GENEHUNTER, which depends on the number of individuals in pedigrees and the number of scanned locations, may present a potential problem to the R program. In the genome scan in this report, the size of the output files is in the range of 6~34 MB. We experienced no problems with the R program in handling these files when running it on a Linux cluster with 512 MB memory on each node. We also tested it on a PC with 256 MB memory and experienced no problems. If the size of the output file from GENEHUNTER is too large for the R program to run, the output file can be split into smaller parts.
